# An integrated assessment of wild vegetable resources in Inner Mongolian Autonomous Region, China

**DOI:** 10.1186/1746-4269-6-34

**Published:** 2010-12-06

**Authors:** Wujisguleng Wujisguleng, Khasbagen Khasbagen

**Affiliations:** 1 Mongolian Medicine College, Inner Mongolia Medical University, Huhhot 010110, China; 2 Inner Mongolia Normal University, Huhhot 010022, China

## Abstract

**Background:**

This paper was based on ethnobotanical investigations conducted from 2004-2006 in Inner Mongolian Autonomous Region of northern China. Today, due to their nutritious and relatively pollution-free characteristics, wild vegetables are playing an increasingly important role in peoples' health and well-being. This paper aims to provide scientific clues for the selection of special and high quality wild vegetables species.

**Methods:**

An ethnobotanical study, consisting of a literature survey, open-ended and semi-structured interviews, and collection and identification of voucher specimens was carried out to gather information on wild vegetables in Inner Mongolia. Next, an integrated assessment of 90 species of wild vegetables was performed using the linearity weighted integrative mathematical analysis method.

**Results:**

According to an integrated assessment of 90 species of wild vegetables in Inner Mongolia, there are 5 species with the highest integrated value, 40 species of high-integrated value, 43 species of general integrated value, and 2 species of low value. The results indicate that the vast majority of wild vegetables have high value in Inner Mongolia.

**Conclusions:**

Inner Mongolia is rich in wild vegetable resources. A comprehensive assessment indicates that the vast majority of wild vegetables are of high value. However, these wild vegetables are seldom collected or cultivated by local people. Most of the collected species require further research and investigation into their nutrient content, toxicity and ethnobotany to illuminate their potential as new cultivars or products.

## Background

### Study Area

Inner Mongolian Autonomous Region (from here on Inner Mongolia), is located in northern China (37°30'~53°20'N, 97°10'~126°02'E) and belongs to the Southeastern Mongolian Plateau, which is in the center of the Asian continent. The total territory is 1.183 million km^2 ^and is the third biggest province/region in China occupying over 12% of China's total land area. Inner Mongolia's geography varies considerably from plateau to mountains, upland, plains, basin and desert. The average altitude is about 1000 m, and consists mostly of plateau, which makes up 53.4% of the province's total area. Mountains account for 20.9% and hilly area for 16.4% of Inner Mongolia's landmass.

Inner Mongolian's climate is temperate continental and characterized by long winters and short summers. The average annual temperature is 6.5°C in the southern and western areas below 0°C in the north and east, and 0-6.5°C in central Inner Mongolia [1,2]. The environment is cooler in the northeastern part of the province and consists mainly of forests and shrub-land, where as in the warmer western area, the environment is made up of mostly grassland and desert. Due to its unique climatic and geologic characteristics, Inner Mongolia has an abundance of wild vegetable resources.

### Aim of study

Vegetables are non-staple foods, which include herbs, woody plants and fungi. Wild vegetables are wild plants used for food but have not yet been introduced, cultivated or managed by people.

Wild vegetables have always been an integral part of the human diet. With the aims of enriching and diversifying the human diet, botanists and agronomists have researched, explored, and cultivated new types of vegetables from all over the world. In order to exploit, utilize, and effectively conserve wild vegetable resources, an integrated assessment is needed. This paper aims to provide scientific clues as to how best to select high-quality species for further exploitation, utilization, and conservation.

### Previous study on wild vegetables

Since the 1950 s, researchers from all over the world have investigated wild vegetable resources and published works on wild edible plants. For example, "Edible Wild Plants of Eastern North America" (1958), " Edible Wild Plants of Eastern United States and Canada" (1976), "A Field Guide to Edible Wild Plants of Eastern and Central North America"(1978), "Wild Green Vegetables of Canada" (1980) etc. [3-6]. In addition to the above studies, there has been much research into the nutritional contents of some key species [7-10]. In the 21^st ^century, the study of wild vegetables is still very active. Recently, botanists have reported on wild vegetable resources in Italy, India and Poland [11-13], and there have been many ethnobotanical studies documenting folk wild vegetables in Africa, Cyprus and Vietnam [14-19].

China's rich history and classical literature on wild vegetables are useful sources of information for researchers. The study of wild vegetable resources has been carried out since the 1950 s in China. Since the 1980 s, ethnobotanical studies have been carried out in Inner Mongolia but have focused on only a few specific species and a limited area [20-27] (see Additional file 1). According to the Flora of Inner Mongolia [1,28-31] (see Additional file 1), 2270 species of vascular plants have been documented in the region. Throughout this investigation, the author has recorded 323 wild vegetables. However, an integrated assessment of wild vegetable resources has not yet been done. In order to effectively use and manage wild vegetable resources, this study was undertaken.

## Methods

Applying standard methods of ethnobotany, our investigation was carried out by the following three steps: literature survey, site selection and field study. The authors used the linearity weighted integrative mathematical analysis method to perform an integrated assessment of 90 species of wild vegetables in Inner Mongolia.

### Literature Surveying

Relevant literature was surveyed and consulted to obtain general information on appropriate wild vegetables. Some information was accessed directly from previous studies on flora [32-43] (see Additional file 1) and wild plant resources [44-49] (see Additional file 1) while some information was obtained indirectly from both domestic [50-65] (see Additional file 1) and international studies on wild edible plants.

### Selecting Sites

In 2004-2006, we selected six local *qi*s (a *qi *or banner in English is a county-level administrative territory) to carry out our investigation in three different vegetation types. The area are as follows: Abag Banner and eastern Uzumqin Banner in Xilingul grassland; Otug Banner, southern Otug Banner, and Hangin Banner in the Ordos shrub-land and sandy vegetation region; and Northern Banner in Tongliao, part of the Horchin sandy area.

### Field Study

Guided by the principles of open-ended interviews and semi-structured interview techniques, we interviewed 98 informants including elderly villagers, Mongolian and Han herbalists, and farmers and herdsmen. From those interviews, we obtained information on wild vegetables, such as timing of edibility, edible parts, and medicinal value. During the field study, we collected specimens of wild vegetables known by the local people.

Plant specimens were examined and identified by the authors. At the same time, we consulted ethnobotanical plant specimens deposited in the herbarium of Inner Mongolia Normal University. For some practical reasons, we have not yet determined nutritional components, toxicity levels, and development status of some species, so the 90 most researched species of the 323 species of wild vegetables were selected for the integrated assessment.

### Integrated Assessment

#### The establishment of integrated assessment index system

**The indicators of the synthetic index system have their own respective importance. In this paper, the following 10 synthetic indices have been used**: nutritional value (NV), distribution (D), community status (CS), life form (LF), basis of civil use (BCU), whether the plant is wild or cultivated or produced (WCP), toxicity (T), edible time (ET), edible parts (EP) and medicinal value (MV). Then the plants were classified and a score was assigned to them (Table 1, Table 2).

**Table 1:** The criteria of division of wild vegetables (mg/kg fresh edible parts)

	Carotene	Vitamin B_2_	Vitamin C
**Type I**	≥ 50	≥ 5	≥ 500

**Type II**	≥ 50	≥ 5	< 500
	≥ 50	< 5	≥ 500
	< 50	≥ 5	≥ 500

**Type III**	≥ 50	< 5	< 500
	< 50	≥ 5	< 500
	< 50	< 5	≥ 500

**Type IV**	< 50	< 5	< 500

**Table 2:** Weight, classification and assignment criteria

Assignment indicator	Weight	Classification	Assignment (score)
NV	0.22	type I	4
		type II	3
		type III	2
		type IV	1

D	0.11	The whole region	3
		Most of the region	2
		Parts of the region	1

CS	0.13	Dominant species	3
		Common species	2
		Rare species	1

LF	0.02	Perennial herb	3
		Annual or Biennial herb	2
		Woody	1

BCU	0.09	wide range	3
		Less	2
		Not	1

WCP	0.02	Cultivation	3
		Gathering and production	2
		Wild	1

T	0.04	None	2
		Low	1

ET	0.13	Cross-seasonal eating	3
		Single-seasonal eating	2
		Short-term eating	1

EP	0.18	More than one	2
		Single	1

MV	0.04	Yes	1
		No	0

Assignment indicator: NV = Nutritional Value; D = Distribution; CS = Community Status; LF = Life Form; BCU = Basis of Civil Use; WCP = whether the plant is Wild or Cultivated or Produced; T = Toxicity; ET = Edible Time; EP = Edible Parts; MV = Medicinal Value.

#### Weight Determination

Weight determination was based on the "function-driven" principles of the set-value iteration method. According to the relative importance of each indicator, the weight of each indicator can be determined. Generally, the means of determination are divided into objective and subjective means [66,67] (see Additional file 1). This paper applied the subjective weighting method.

We first made five index sub-sets by consulting with five relevant experts from Inner Mongolia Normal University. Each experts randomly chose *S *= 3 indices at the first step and chose *S *= 2 × 3 indices at the second step, which were considered more important for this study of 10 indices, followed by the creation of index sub-sets. In order to facilitate selection, each indicator is numbered as follow: ① NV, ② D, ③ CS, ④ LF, ⑤ BCU, ⑥ WCP, ⑦ T, ⑧ UT, ⑨ UP, ⑩ MV. Index sub-sets created by the five experts are as follows:

*Expert 1 *: (1) { ① ② ③}, (2) { ① ② ③ ⑤ ⑨ ⑩}

*Expert 2 *: (1) { ① ③ ⑨}, (2) { ① ③ ⑤ ⑦ ⑧ ⑨}

*Expert 3 *: (1) { ① ⑧ ⑨}, (2) { ① ② ③ ⑧ ⑨ ⑩}

*Expert 4 *: (1) { ① ⑤ ⑨}, (2) { ① ② ③ ⑤ ⑥ ⑧}

*Expert 5 *: (1) { ① ⑧ ⑨}, (2) { ① ② ④ ⑦ ⑧ ⑨}

After making five index subsets, the weight (*w_j_*) of each indicator was calculated using the following formula.

(1) $$g(x_{j})=\sum_{k=1}^{5}\sum_{i=1}^{2}u_{ik}(x_{j}),j=1,2,\cdot \cdot \cdot ,\text{10}$$

To

(2) $${\text{u}}_{}(x_{j})={\{}_{\text{0, }x_{j}\notin {\text{X}}_{i,k}}^{\text{1, }x_{j}\in {\text{X}}_{i,k}}(i=1,2;k=1,2,\cdot \cdot \cdot ,5)$$

In Formula 1, *x_j _*is the total number of times the *j*-st index was selected. *k *is the number of experts, and *i *is the number of selecting steps. *u_ik _*(*x_j_*) is defined as whether the *j*-st index is selected by the *k*-st expert in each index subset, and *X_i,k _*in Formula 2 is the index subset reported by the *k*-st expert at the *i*-st step.

After normalization of *g*(*x_j_*), the weight (*w_j_*) corresponding to index *x_j _*was calculated by using Formula 3. The results are shown in Table 1.

(3) wj=g(xj)∑k=110g(x)k,j=1,2,⋅⋅⋅,10

#### Integrated Assessment

An integrated assessment was developed by the linearity weighted integrative mathematical analysis method. This method is the application of the weighted linear model (Formula 4) to conduct a comprehensive evaluation of plant resources. The integrated value of each species is calculated by using Formula 4 Where *y *is the integrated value of wild vegetable, *w_j _*$\left(0\leq w_{j}\leq 1(j=1,2,\cdot \cdot \cdot ,10),\sum_{j=1}^{10}w_{j}=1\right)$ is the weight corresponding to index *x_j_*, and *x_j _*(*j *= 1,2, ..., 10) are the indices:

(4) $$y=\sum_{j=1}^{10}w_{j}x_{j}$$

Their scores are shown in Table 3.

**Table 3:** Integrated value to parts of the wild vegetables in Inner Mongolian Autonomous Region

Latin name of wild vegetable	NV	D	CS	LF	BCU	WCP	T	ET	EP	MV	IV
*Sanguisorba officinalis *L.	4	2	3	3	2	2	1	3	2*	1	2.68
*Polygonum aviculare *L.	4	3	2	2	3	2	1	3	2*	1	2.65
*Potentilla anserina *L.	3	3	3	3	3	2	1	3	2*	1	2.58
*Platycodon grandiflorus *(Jacq.) A. DC.	4	2	2	3	3	2	1	3	2**	1	2.56
*Vicia amoena *Fisch.	4	2	2	3	2	2	2	3	2*	1	2.55
*Hemerocallis minor *Mill.	3	2	3	3	3	3	1	3	2*	1	2.49
*Sonchus arvensi*s L.	3	3	2	3	3	3	1	3	2*	1	2.47
*Taraxacum mongolicum *Hand.-Mazz.	3	3	2	3	3	3	1	2	2*	1	2.47
*Polygonum divaricatum *L.	4	2	2	3	1	1	1	2	1*	1	2.47
*Adenophora trachelioides *Maxim.	4	2	2	3	1	1	2	3	2**	1	2.45
*Potentilla fruticosa *L.	3	2	3	1	1	1	1	2	1*	1	2.44
*Thalictrum minus *L. var. *hyploeucum *(Sieb. et Zucc.) Miq.	4	2	2	3	1	1	1	2	2*	0	2.43
*Agrimonia pilosa *Ledeb.	4	2	2	3	1	1	1	3	2*	1	2.38
*Vicia unijuga *R. Br.	4	2	2	3	1	1	1	3	2*	1	2.38
*Viola acuminata *Ledeb.	4	2	2	3	1	1	1	3	2*	1	2.38
*Chenopodium album *L.	3	3	2	2	3	2	1	2	2*	1	2.37
*Calystegia hederacea *Wall. ex Roxb.	4	2	2	2	1	1	1	3	2*	1	2.36
*Eleutherococcus senticosus *(Rupr. et Maxim.) Maxim.	4	1	2	1	2	2	1	3	2*	1	2.35
*Vicia cracca *L.	4	2	2	1	1	1	1	3	2*	1	2.34
*Sonchus oleraceus *L.	3	2	2	2	3	2	1	3	2*	1	2.32
*Potentilla supina *L.	4	3	2	2	1	1	1	2	2*	0	2.30
*Plantago asiatica *L.	2	3	3	3	2	2	1	3	2*	1	2.28
*Trifolium lupinaster *L.	4	2	2	3	1	1	1	2	2*	1	2.25
*Solanum nigrum *L.	3	2	2	2	2	1	1	3	2*	1	2.22
*Portulaca oleracea *L.	2	3	2	2	3	2	1	3	2*	1	2.21
*Malva verticillata *L.	3	3	2	2	3	2	1	3	1*	0	2.21
*Actaea dahurica *Turcz.	3	2	2	3	1	2	1	3	2*	0	2.18
*Artemisia selengensis *Trucz. ex Bess.	2	2	3	3	2	2	1	3	2*	1	2.17
*Lysimachia barystachys *Bunge	3	2	2	3	1	1	1	3	2*	1	2.16
*Matteuccia struthiopteris *(L.) Todaro	3	2	3	3	1	2	1	3	1*	1	2.13
*Codonopsis lanceolata *(Sieb. et Zucc.) Benth. et Hook. f.	3	2	1	3	2	2	1	3	2**	1	2.13
*Polygonum lepathifolium *L.	3	3	2	2	1	1	1	2	2*	1	2.12
*Polygonatum odoratum *(Mill.) Druce	3	2	2	3	2	1	1	2	2**	1	2.11
*Polygonum hydropiper *L.	3	2	2	2	2	1	1	2	2*	1	2.09
*Allium senescens *L.	2	2	2	3	3	2	1	3	2*	0	2.08
*Allium ramosum *L.	2	2	2	3	3	2	1	3	2**	0	2.08
*Allium macrostem*on Bunge	2	2	2	3	2	3	1	3	2**	1	2.06
*Thalictrum squarrosum *Steph. ex Willd.	3	2	2	3	1	2	1	2	2*	1	2.05
*Melilotus suaveolens *Ledeb.	4	2	2	2	1	1	1	2	1*	1	2.05
*Mentha haplocalyx *Briq.	2	2	2	3	2	2	1	3	2*	1	2.04
*Lagedium sibiricum *(L.) Sojak	2	2	2	3	1	2	2	3	2*	1	2.03
*Thalictrum aquilegifolium *L. var. *sibiricum *Regel et Tiling	4	2	2	3	1	1	1	2	1*	0	2.03
*Capsella bursa-pastoris *(L.) Medic.	2	2	2	2	1	1	1	3	2*	1	2.02
*Kochia scoparia *(L.) Schrad	2	3	2	2	2	2	1	2	2*	1	2.00
*Salsola collina *Pall.	2	3	2	2	2	2	1	2	2*	1	2.00
*Amaranthus retroflexus *L.	1	3	2	2	3	2	1	3	2*	1	1.99
*Nymphoides peltata *(S. G. Gmel.) Kuntze	2	3	2	3	1	1	2	3	1*	1	1.99
*Cardamine leucantha *(Tausch) Schulz	3	2	1	3	1	1	1	3	2*	0	1.99
*Monochoria vaginalis *(Burm.) Presl.ex Kunth	3	2	1	1	1	1	1	3	2*	1	1.99
*Rorippa globosa *(Turcz.) Thell.	3	2	2	2	1	1	1	2	2*	0	1.97
*Suaeda salsa *(L.) Pall	2	2	3	2	2	1	1	2	2*	0	1.96
*Lycium chinense *Mill.	3	1	1	1	2	3	2	3	2*	1	1.95
*Lycopus lucidus *Turcz. ex Benth.	2	2	2	3	1	1	1	3	2**	1	1.94
*Pedicularis resupinata *L.	2	2	2	3	1	1	1	3	2*	1	1.94
*Ixeris chinensis *(Thunb.) Nakai.	1	3	2	3	2	2	1	3	2*	1	1.93
*Potentilla bifurca *L.	3	3	2	3	1	1	1	2	1*	0	1.92
*Lathyrus davidii *Hance	4	1	1	3	1	1	1	3	1*	0	1.92
*Rumex acetosa *L.	2	2	2	3	2	2	1	2	2*	1	1.91
*Viola verecunda *A. Gray	3	1	1	3	1	1	1	3	2*	0	1.91
*Ulmus pumila *L.	1	3	2	1	3	1	1	1	2***	1	1.91
*Plantago major *L.	2	2	2	3	1	1	1	3	2*	0	1.90
*Patrinia scabiosaefolia *Fisch. ex Trev.	3	2	2	3	1	1	1	1	2*	1	1.90
*Galinsoga parviflora *Cav.	3	1	1	2	1	1	1	3	2*	1	1.90
*Kummerowia striata *(Thunb.) Schindl.	4	1	2	2	1	1	1	2	1*	0	1.90
*Allium mongolicum *Regel	1	2	2	3	3	3	1	3	2*	0	1.88
*Arctium lappa *L.	2	2	2	2	1	1	1	3	2**	0	1.88
*Glaux maritima *L.	2	3	3	3	1	1	1	3	1*	0	1.86
*Asparagus schoberioides *Kunth	3	2	2	3	1	1	1	2	1*	1	1.85
*Medicago lupulina *L.	3	2	2	2	1	2	1	2	1*	1	1.85
*Pteridium aquilinum *(L.) Kuhn var. *latiusculum *(Desv.) Underw. ex Heller.	1	2	2	3	2	3	1	3	2*	1	1.84
*Athyrium multidentatum *(Doell) Ching	3	1	2	3	1	1	1	3	1*	0	1.83
*Sedum aizoon *L.	2	2	1	3	1	1	1	3	2*	1	1.81
*Carduus crispus *L.	1	3	2	2	1	1	1	3	2*	1	1.81
*Schisandra chinensis *(Turcz.) Baill.	2	2	1	1	2	1	1	3	2*	0	1.81
*Allium tenuissimum *L.	1	2	2	3	2	2	1	3	2*	0	1.78
*Kummerowia stipulacea *(Maxim.) Makino	4	2	2	2	1	1	1	2	1*	1	1.75
*Lespedeza caraganae *Bunge	4	1	1	1	1	1	1	2	1*	0	1.75
*Cirsium segetum *Bunge	2	3	2	3	1	1	1	2	1*	1	1.74
*Oxalis corniculata *L.	3	1	1	3	1	1	1	3	1*	1	1.74
*Typha latifolia *L.	1	2	2	3	1	1	1	3	2*	1	1.72
*Sanicula chinensis *Bunge	2	1	1	3	1	2	1	3	2*	0	1.68
*Rheum franzenbachii *Munt.	3	2	1	3	1	1	1	2	1*	0	1.68
*Atractylodes japonica *Koidz. ex Kitam.	2	1	1	3	1	1	1	3	2*	0	1.66
*Commelina communis *L.	2	2	2	2	1	1	1	2	1*	1	1.63
*Kalimeris integrifolia *Turcz.ex DC.	1	2	2	3	1	2	2	2	1*	1	1.60
*Syneilesis aconitifolia *(Bunge) Maxim.	1	2	2	3	1	1	1	2	2*	1	1.59
*Conyza canadensis *(L.) Cronq.	3	1	1	2	1	1	1	2	1*	1	1.59
*Suaeda glauca *(Bunge) Bunge	1	2	3	2	2	1	1	2	1*	0	1.56
*Ulmus macrocarpa *Hance	1	2	3	1	2	1	1	1	2***	0	1.49
*Oenanthe javanica *(Bl.) DC.	1	1	1	3	2	1	1	3	1*	0	1.26

EP(Edible Parts): * green vegetative part (leaf and stem); ** green vegetative part (leaf and stem) and underground part (roots and rhizomes); *** green vegetative part (leaf and stem) and green fruit.

Obtained by combined with the data in Table 2, Formula 4 is shown below. The integrated value of the wild vegetables was calculated, and the results are shown in Table 3.

IV=0.22×NV+0.11×D+0.13×CS+0.02×LF+0.08×BCU+0.02×WCP+0.07×T+0.13×ET+0.18×EP+0.04×MV

## Results and Discussions

### Single analysis to indices

#### Nutritional value

In this paper, three types of vitamins (carotene, vitamin B_2 _and vitamin C) were selected to illustrate and reflect the nutritional value of wild vegetables. Considered from their vitamin standpoint, 21(23.33%) species are type I wild vegetables, 31 (34.44%) are type II, 25 (27.78%) are type III and 13 (14.44%) are type IV [64].

#### Distribution, community status and life form

The majority of wild vegetables are distributed throughout Inner Mongolia and where as the rest are found in a very small area. As for the community status of the 90 species surveyed, 63 species (70%) are common, 11 (12.22%) are rare, and 16 (17.78%) make up the dominant vegetation. Fifty-six plants, over half of the species, are perennial herbs, while 25 are annual or biennial herbs and only nine are woody. These conditions directly influence the identification, collection and consumption of wild vegetables.

#### Toxicity

Toxicity levels of wild vegetables in Inner Mongolia are divided into three categories. The first level indicates that the toxic elements of the plant are only present during certain times of the plants' life cycle. The second level indicates that people may be poisoned or sickened by over eating or prolonged ingestion of the toxic plant. The third level indicates that poisonous elements of the plant can be removed by processing. The majority of wild vegetables contain small amounts of toxic chemical substances, but all of them are edible after processed. At present, commonly used methods to remove toxicity are to soak the edible parts in cold water or to blanch them in boiling water. The Mongolians have a unique way to remove poisonous elements in wild vegetables by cooking and eating the edible parts in milk. Because of the prevalence of toxic elements in wild vegetables, we must fully understand how to safely ingest the plant by investigating the period of edibility, edible parts, edible dosages, and processing methods of wild vegetables before regarding them as fully safe.

#### Basis of civil use and whether the plant is wild, cultivated or produced

The ethnobotanical survey indicated that 15 (16.67%) species are widely gathered and used, 23 (25.56%) are seldom known or used by the local people, and 52 (57.78%) have not been used. Among the widely used species, *Allium mongolicum *Regel, *A. macrostemon *Bunge, *Sonchus arvensis *L., *Hemerocallis minor *Mill., *Taraxacum mongolicum *Hand.-Mazz. have been cultivated. Other cultivated species include *Sanguisorba officinalis *L., *Polygonum aviculare *L., *Potentilla anserina *L., *Potentilla anserina *L., *Platycodon grandiflorus *(Jacq.) A. DC., and *Vicia amoena *Fisch.. The remaining species are still fully wild and have not yet been cultivated.

#### Edible parts and edible time

Sixty-six (73.33%) wild vegetables are harvested for more than one part of the plant. These various parts include the leaves or upper parts (leaf and stem), green fruits and underground parts (roots and rhizomes). Twenty-four (26.67%) are harvested for a single part. Fifty-six (62.22%) are available in multiple seasons, 31 (34.44%) are only available during a single-season and only three (3.33%) are available for less than one season.

#### Medicinal value

According to field study and literature review, the author found that 62 out of 90 (68.89%) species of wild vegetables are used as Mongolian medicine or Chinese medicine.

### Integrated value

Based on integrated value shown in Table 3, the wild vegetables in Inner Mongolia were divided into 4 grades: highest, high, general and low (Table 4).

**Table 4:** Integrated value of the evaluation criteria

Integrated value	>2.5	2.0-2.5	1.5-2.0	<1.5
**Grade**	highest	high	general	low

**Figure 1:**
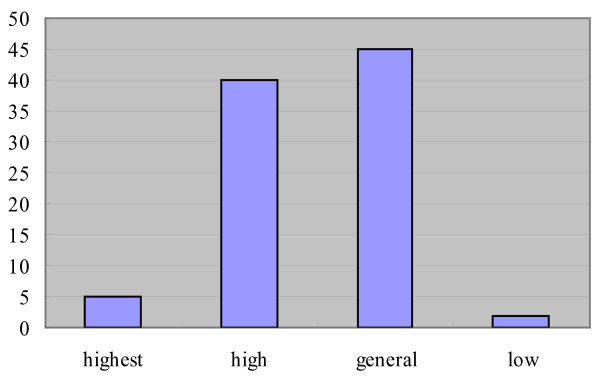
**Integrated value grade of wild vegetables in Inner Mongolia**.

There are 5 species with the highest value (integrated value > 2.5), 40 species with high value (2.0 < integrated value < 2.5), 43 species with general value (1.5 < integrated value < 2.0) and only two species with low value. Ninety percent or 83 species have a high or general value. The highest and low value wild vegetables are few in Inner Mongolia (Figure 1).

Indicated from the single analysis to the indices, woody plants, annual or biennial herbs, species distributed in small parts, rare species, and species with short edibility time or small reserves, have lower integrated values. Because of the above traits, they are seldom known or used by the local people and have not been cultivated. But in fact, these wild vegetables, including *Commelina communis *L., *Rheum franzenbachii *Munt., *Kummerowia stipulacea *(Maxim.) Makino, etc., possess high nutritional and medicinal value and little toxicity.

The integrated assessment indicates that due to their low nutritional value, small edible part, and short edible time, some of the most popular wild vegetables in Inner Mongolia as *Amaranthus retroflexus *L., *Ulmus pumila *L., *Suaeda glauca *(Bunge) Bunge show a lower integrated value. In addition, local people less frequently know and eat some species that possessing the highest or higher integrated values. *Sanguisorba officinalis *L., *Platycodon grandiflorus *(Jacq.) A. DC., *Vicia amoena *Fisch., *Adenophora trachelioides *Maxim., *Agrimonia pilosa *Ledeb., *Vicia unijuga *R. Br. are examples of species that despite their high-integrated value are infrequently used by local people. Whether considered from a nutritional standpoint or from their biological and ecological traits, all of the above plants possess higher edible value and characteristics of easy cultivars, but within the communities surveyed there is little awareness of them.

## Conclusions

Inner Mongolia is rich in wild vegetable resources, but the majority of them are seldom collected or cultivated because of their biological and ecological traits. Traits that contribute to the uncommon usage of these wild vegetable resources include life form, life cycle, distribution, abundance, edibility time, size of plant and toxicity.

According to the integrated assessment, the key species in Inner Mongolia were discovered. These include, *Sanguisorba officinalis *L., *Polygonum aviculare *L., *Potentilla anserina *L., *Platycodon grandiflorus *(Jacq.) A. DC., *Vicia amoena *Fisch etc.. These species exhibit the traits of high-quality vegetables, such as high vitamins content, no toxicity or have toxins that are easy to remove, unique taste, appropriate edible parts, suitability for cultivation, simple to collect and process, and so on. The species with the above traits are worthy of further research and development.

The studies of wild vegetable resources in Inner Mongolia are only beginning. In order to introduce new products and increase dietary diversity of local people, complimentary studies and further ethnobotanical studies should be performed. The enormous amount of traditional knowledge and understanding of wild vegetables could be very useful for management strategies and life-style choices for local people. This knowledge and understanding may serve as baseline data for future development. In order to ensure the nutritional and toxicity content of the wild vegetables, a detailed determination of nutritional and poisonous components should be conducted. Finally, further comprehensive assessments, measuring the integrated values of wild vegetables should be performed to further understand their potential for cultivation or product development.

## Competing interests

The authors declare that they have no competing interests.

## Authors' contributions

WW conducted field surveys and interviews with the local people, identified the herbarium with KK, drafted and finalized the manuscript with KK. KK supervised the research works. All authors have read and approved the final manuscript.

## Supplementary Material

Additional file 1 **Appendix 1**. References from China in Chinese Click here for file
