# Supplementary material for: An integrated assessment of wild vegetable resources in Inner Mongolian Autonomous Region, China
Source: J Ethnobiol Ethnomed. 2010 Dec 6;6:34. doi: 10.1186/1746-4269-6-34 (PMC3009950; doi:10.1186/1746-4269-6-34)
Supplement: Additional file 1 — Appendix 1. References from China in Chinese [file 1746-4269-6-34-S1.DOC]

Appendix 1

References from China in Chinese

1. 中国科学院内蒙古宁夏综合考察队. 内蒙古植被

2. 马毓泉. 内蒙古植物志（第二版，第一卷）

21. 斯琴巴特尔，刘新民. 蒙古韭的营养成分及民族植物学.中国草地

22. 耿星河. 内蒙古庭院8种常见野菜的营养成分分析.内蒙古师范大学学报（自然科学汉文版）

23. 曹晓明.沙区食用植物—沙芥的开发利用.中国水土保持

24. 金凤，哈斯巴根.反枝苋的研究现状与开发利用.内蒙古师范大学学报，自然科学（蒙文版）

25. 乌尼尔，哈斯巴根.内蒙古呼伦贝尔鄂温克族民间野菜资源调查.中国野生植物资源

26. 哈斯巴根.《蒙古秘史》中的野生食用植物的研究.干旱区资源与环境

27. 乌吉斯古楞，哈斯巴根.内蒙古蒙药蔬菜兼用野生植物的初步研究.民族植物学与药用植物

28. 马毓泉.内蒙古植物志（第二版，第二卷）

29. 马毓泉.内蒙古植物志（第二版，第三卷）

30. 马毓泉.内蒙古植物志（第二版，第四卷）

31. 马毓泉.内蒙古植物志（第二版，第五卷）

32. 中国科学院中国植物志编辑委员会.中国植物志（第十四卷）

33. 中国科学院中国植物志编辑委员会.中国植物志（第二十五卷第二分册）

34. 中国科学院中国植物志编辑委员会.中国植物志（第三十三卷）

35. 中国科学院中国植物志编辑委员会.中国植物志（第六十三卷）

36. 刘媖心.中国沙漠植物志（第一卷）

37. 刘媖心.中国沙漠植物志（第二卷）

38. 刘媖心.中国沙漠植物志（第三卷）

39. 毛子军，王秀华，穆丽蔷,等.黑龙江植物志（第八卷）

40. 聂绍荃，张艳华.黑龙江植物志（第六卷）

41. 张桂一，袁晓颖，李兆华,等.黑龙江植物志（第九卷）

42. 董世林，等.黑龙江植物志（第四卷）

43. 西北植物研究所.黄土高原植物志（第一卷）

44. 胡先骕.经济植物手册（上册）

45. 胡先骕.经济植物手册（下册，第一份册）

46. 中国科学院林业土壤研究所.东北资源植物手册

47. 中华人民共和国商业部土产废品局，中国科学院植物研究所.中国经济植物志（上、下册）

48. 中国科学院西北高原生物研究所.青海经济植物志

49. 李惠民.山西省经济植物志

50. 洪学智.东北野生可食植物

51. 董淑炎，魏宗荣，杨成俊.中国野菜食谱大全

52. 赵玉平.中国温带野菜加工工艺

53. 卢炯林，张俊朴，苏金乐，等.河南野菜野果

54. 樊守金，赵遵田. 山东野生蔬菜志

55. 董然，李广巨，樊绍林.长白山野菜

56. 张哲普.野菜的食用及药用

57. 高愿军.中国野生植物开发与加工利用

58. 徐道东，赵章忠，王统正，等.多年生与野生蔬菜栽培技术

59. 郭文场.野菜栽培与食用

60. 杨毅，傅运生，王万贤.野菜资源及其开发利用

61. 徐启国，杨柏明.食药兼用野菜的栽培与利用

62. 许又凯，刘宏茂.中国云南热带野生蔬菜

63. 徐践.野菜栽培与加工

64. 陶桂全，郭志成，李新加，等.中国野菜图谱

65. 赵金光，韦旭斌，郭文场.中国野菜

66. 郭亚军.综合评价理论与方法

67. 胡永宏，贺思辉.综合评价法
